# A novel investigation of the influence of corporate governance on firms’ credit ratings

**DOI:** 10.1371/journal.pone.0250242

**Published:** 2021-05-04

**Authors:** Abdullah A. K. Alkhawaldeh, Jamil J. Jaber, Dalila Boughaci, Noriszura Ismail

**Affiliations:** 1 Department of Accounting, Faculty of Economics and Administrative Sciences, The Hashemite University, Zarqa, Jordan; 2 Department of Risk Management and Insurance, The University of Jordan, Aqaba Branch, Aqaba, Jordan; 3 Computer Science Department, University of Science and Technology Houari Boumediene, FEI, Algiers, Algeria; 4 National University of Malaysia, School of Mathematical Sciences, Bangi, Malaysia; DePaul University, UNITED STATES

## Abstract

Corporate governance is the way of governing a firm in order to increase its accountability and to avoid any massive damage before it occurs. The aim of this paper is to investigate the impact of capital structure, firms’ size, and competitive advantages of firms as control variables on credit ratings. We investigate the role of corporate governance in improving the firms’ credit rating using a sample of Jordanian listed firms. We split firms into four categories according to WVB credit rating. We use both the binary logistic regression (LR) and the ordinal logistic regression (OLR) to model credit ratings in Jordanian environment. The empirical results show that the control variables are strong determinants of credit ratings. When we evaluate the relationship between the governance variables and credit ratings, we found interesting results. The board stockholders and board expertise are moderately significant. The board independence and role duality are weakly significant, while board size is insignificant.

## 1 Introduction

Creditworthiness is the ability of a given corporation to fully meet its financial obligations as they come due. For instance, when a loan applicant applies for credit or funds, lenders will check its credit score to determine whether it can get credit or not. Therefore, companies have to find a system which would guarantee the non-default of such applicants. As a result of this, corporate governance may be a suitable solution to this problem expressing the organized relationship between managers and shareholders of the corporation.

Corporate governance refers to the way a corporation is governed in order to expand its accountability and to avoid any massive damage before it occurs. Moreover, corporate governance can be viewed as a set of principles and strategies that identify the interaction between various participants in the corporation (such as the board of directors, managers, creditors, shareholders, regulators, auditors, and other stakeholders) and include the rules and procedures for making decisions in corporate affairs.

Since the role of corporate governance is prominent, various studies have attempted to empirically examine the relationship between corporate governance and various aspects. Among them, we give the following ones: the studies on the firm performance [1–3], the studies on the firm value [4–6] and the studies on the impact of corporate governance on credit rating [7–9]. Other studies have investigated a corporation’s credit rating by relating it to various variables, such as the firm’s characteristics and debt’s cost [10]. In addition, the authors in [11] have studied the impact of corporate governance on firm’s value in Thailand. A similar study is conducted in [12] where an empirical analysis is done on the impact of corporate governance on some firms from Iran. In [13], the authors have studied the effects of board and ownership structure on corporate performance. In [14], the authors have examined the impact of ownership structures on Indonesia companies. The authors in [15] have studied the impact of firm ownership structure on corporate social responsibility: evidence from austerity U.K. In [16], authors have studied the influence of firm efficiency on agency credit ratings using a sample of Korean listed firms.

Almost all of the studies on corporate governance tend to focus on one variable, such as, board independence, as opposed to studying the board sets of governance variables intended to protect stakeholders’ claims on firms’ resources [7, 8]. In [9], authors suggest that some variables may complement each other in supporting stakeholders, whereas other governance factors may be regarded as substitutes. Consequently, only governance variables may give rise to the problem of omitted variables.

The selection of appropriate governance variables that can be evaluated is an important issue. In this study, we focus on the variables that can represent the key features of good governance practices for Jordanian firms. We consider eight independent variables: three variables as control variables and five variables as corporate governance. The control variables are: the leverage, the firm size and the growth opportunity (Tobin’s *q*). It is one of several financial measurements that look at how much capital comes in the form of debt (loans) or assesses the solvency ratio that indicates whether a company’s cash flow is sufficient to meet its short-and long-term liabilities. The firms’ size is a very important variable for stakeholders because it measures the ability of firms to cover its obligation after liquidation. Liquidation occurs when a company becomes insolvent, meaning that it cannot pay its obligations when they come due. In the event of liquidation, the creditors get first priority to recover their money, and the shareholders take the residuals. Tobin’s *q* is widely used to determine the competitive advantages of a company. It concentrates on what the firm is worth today relative to what it would cost to replace it today. High ratio tends to attract investment opportunities or significant competitive advantages of companies. If the ratio is greater than 1, it reflects the overvalued company and indicates that a company’s earnings are higher than the assets’ replacement costs. On the other side, the company is undervalued if the ratio is lower than 1. In this case, the company may be attractive to potential purchasers who would be willing to buy the company instead of creating a similar company. The company is fairly valued if the ratio equals 1. The corporate governance variables are: the board independent directors, the role duality, the board expertise, the board stock and the board size. We use both the logistic regression (LR) and the ordinal logistic regression (OLR) models to analyze our data. Marginal effects are also investigated for dichotomous credit rating categories. We conduct empirical analysis to examine the relationship between the considered corporate governance variables and the firms’ credit ratings. We take as samples the Jordanian firms listed in the Amman Stock Exchange (ASE), available at the link: https://www.ase.com.jo/. We examine the level of credit rating practices in Jordan from 2015 to 2017.

The main contributions of this study can be summarized as follows. First, according to the theory dimension, this research work extends the extant literature on credit rating by focusing on how important theories, such as those pertaining to corporate governance, impact on credit rating. However, only few studies [7–9] have discussed the application of such theories to credit rating in a developed country context. These theories enrich the research in the area of credit rating by adding supportive power to the explanation of using factors which have an influence on the credit rating. Such a power can raise the awareness of the current credit rating situation as a subject of one of the developing countries with unique political and economic characteristics. Second, the investigation of corporate governance in the Jordanian environment is another contribution of this research. Most studies focus on the corporate governance topic in general without attempting to link this topic to the field of credit assessments. Only a few studies have forged a link between corporate governance and credit rating [7–9]. Further, the current research makes a contribution by investigating some new variables not used before in this context of credit rating. These variables are related to company corporate governance variables, such as, the role duality and the board size. Moreover, this research presents the effects of control variables with governance variables on credit rating.

The rest of this paper is organized as follows. Section 2 presents the motivations for our work. Section 3 gives a review of the relevant literature and develops hypotheses. Section 4 details the research design and the techniques used in this study. Section 5 reports the results of the empirical analysis. Finally, Section 6 concludes and presents some future works.

## 2 Our motivations

The current research is based on data from Jordanian firms listed on the Amman Stock Exchange (ASE) as an emerging capital market. Most studies addressing credit rating are applied to developed countries. By contrast, the present work has sought to study the impact of credit ratings in a developing country, namely, Jordan.

The important features of the Jordanian market corporate governance and credit rating that the Jordanian firms are following the corporate governance code and international accounting standards (IAS), the last over two decades. Jordan creates an attractive investment climate transformed into a modern capital market with a new legal framework and applies the code of corporate governance and IAS. Jordan joined early trade agreements such as the free trade agreement with the EU and USA which need to link the requirements of corporate financial reporting with IAS.

In 2008 the market capitalization to Gross Domestic Product (GDP) ratio was about 26.3 percent, this ratio is one of the highest ratios in the world, and it is one of the largest stock markets in the region that permits foreign investment. From 2009 ranks Jordan in second or third quintile of the global indices benchmarking for political, economic, business, and human capital dimensions. Since the last two decades, Jordanian corporations apply principles of corporate governance, consistent with western countries. We can say that today, the Jordanian firms have developed greatly and succeeded in accomplishing several of its goals by mobilising capital into the productive sectors of the economy [17].

Motivated by these considerations, we investigate the impact of corporate governance on Jordanian firms’ credit ratings. We believe that good corporate governance practices in Jordan may provide a means of enhancing its competitiveness to attain a high level of credit rating. We used the World Vest Base (WVB) as a credit rating agency.

World’vest Base Inc CRA is a credit rating agency initially founded in 1985 in the USA with subsequent offices in Hong Kong, Vietnam, Egypt, Mexico, Thailand, China and so on. The agency is reorganized under the Financial Intelligence Service Ltd. WVB provides several ratings worldwide and employs a strong workforce. Since 1997, WVB has provided data directly to customers and through other distributors, such as Bureau van Dijk Publishing. The WVB rate is an indicator of credit rating that can be used to measure the probability of default of firms. The institution takes micro and macro variables to compute the probability of defaults and then classifies the firms as WVB rates. This credit rate reflects the probability of default for a given firm. For instance, if a firm gets a *D* score (according to WVB rating agency) that means it has a high probability of default and it can’t meet its obligation at time and it has lower efficiency. We can say that it has high credit risk. On the other hand, if a firm gets *BB* score that means it has lower probability of default and it can meet its obligation at time and it has high efficiency. In such a situation, we can say it has low credit risk. WVB still has a strong focus on the Asian and Middle East market. WVB created value-added products in the fields of credit and business risk rating, and analysis, and has an investment evaluation and consultancy service. We used WVB rating because it is commonly used in Jordanian companies. And, the data is available.

All the results of our proposed models enrich our understanding of credit rating assessments in Jordan. In this study we demonstrate that the World Vest Base (WVB) score analysis shows that there are differences in the impact of variables on credit ratings. Thus, this research has enabled deeper insights to be gained into credit rating determinants within the Jordanian context.

## 3 Literature review and hypotheses

In this section, we review relevant literature and develop hypotheses.

### 3.1 Corporate governance and its relationship with credit ratings

Several studies have expounded the concept of corporate governance. Among them we give the following ones. We start with the well-known UK report from the Cadbury Committee (1992 and 2004). The Cadbury Report is titled Financial Aspects of Corporate Governance. This report gives some guidelines and recommendations on the arrangement of company boards and accounting systems to mitigate risks and failure [18]. Further, the study of Blair shows that corporate governance is the set of legal, cultural and institutional arrangements that determine what publicly traded companies can do. In [19], the authors give the principles of best practice of corporate governance and show that these principles are congregating worldwide and each country only differentiates itself in applying corporate governance. Also, it shows who controls them, how the control is exercised and how the risk and returns from the activities they undertake are allocated [20].

As already discussed, corporate governance has an important role on the firm performance. Corporate governance plays an important role in regulating relationships between bondholders and shareholders through better monitoring of management [9]. Some studies have shown that certain governance mechanisms can have a negative impact on bondholders. For example, this can happen when some shareholders are more likely to obtain more power in their hands [21]. Also, certain parties of a company may exercise influence over the management of the company by obtaining priority in the distribution payments and preferential treatment of other parts of the company, such as shareholders, by dividend or share re-purchase or through greenmail to increase in stock prices for avoiding unwanted takeover [22]. As a result, the corporate governance structure can give shareholders more power to influence managers in their decisions to invest in riskier projects and to make changes in ownership structure which can lead to a negative effect on bondholders’ interests. This means that the voting right of shareholders may adversely affect bondholders, by default, their expected future cash flows, as well as the company by its lower risk profile, which may result in a lower creditworthiness. Shareholder rights, which affect the transfer of wealth from bondholders to themselves as a result of shareholder practices, determine whether companies, for example, will approve a merger or acquisition based on shareholder interests with potentially harmful effects on bondholders. This negatively affects bondholders by reducing their benefits. This can increase the default risk of bondholders and the credit rating [9, 23].

### 3.2 Development of hypotheses

In this section, we give and explain the different hypotheses related to the corporate governance variables.

#### 3.2.1 Independent of directors

Board independence is one of the main board of directors’ characteristics. To increase the performance and efficiency of the board of directors, a majority of members should be independent [24]. We note that the existence of independent directors in the board of directors of a firm will restrict opportunism by the managers of the firm and will improve the effectiveness of such a firm [25]. According to the agency theory, the board of directors of a firm should include a majority of outside directors. These members are independent of management and are more willing to effectively oversee management. In addition, the stewardship theory suggests that control should remain in the hands of internal managers, as there is no need for independent oversight mechanisms for those considered trustworthy and committed. In our case in Jordan, the corporate governance code recommends that the board should include at least one-third of independent board members where the aim is to ensure an independent presence and voice on the board. The legal definition of independent directors needs that they have not previously been employees of the corporation, that they have no family or professional relationship with them, that they do not hold executive positions, that they do not represent significant shareholders and have not worked in the company for three years. Consequently, the relationship between the board independence and credit ratings is sketched in the following hypothesis:

*Hypothesis—1: Board independence does not affect the credit ratings for the Jordanian firms*.

#### 3.2.2 Role duality

The role duality refers to two positions at the same time for the same person where the positions are the chief executive officer (CEO) and the chair of the board. The CEO has a full-time position. He is responsible for the management of the firm. However, the chairman is usually part-time where the main responsibility is to ensure the effectiveness of the board [26]. We note that all the best practice codes encourage the separation of the two positions which lead to a more independent board [18]. This separation of the two positions makes it easier for managers to exert self-interested behavior without any control over them [27]. For our case, the Jordanian code calls for a balanced board, so that too much power is not vested in any one individual, which may compromise the quality of the board’s decision making. Consequently, the separation of the roles of the CEO and chairman is recommended, so as to avoid concentration of power, and to serve as a balancing mechanism for the board. The hypothesis is as:

*Hypothesis -2: Role duality does not affect the credit ratings for the Jordanian firms*.

#### 3.2.3 Board expertise

The board expertise refers to some directors within the board sitting on more than one board at the same time [8]. This facilitates the transition experiences between these boards and should allow them to share information in order to collect the highest rating. The availability of directors or chairs of more than one board of directors may affect the credit rating because of the transition experience acquired by other companies. According to the agency’s theory, with respect to the conflicts between the roles of directors sitting on more than one board, this conflict should have an impact on the credit rating, because one person sitting on more than one board will affect board decisions due to the influence and power of this person through transferring this experience from one corporation to another. However, several studies have argued that the expertise and knowledge of board experts allows for better management control and better decision-making, as well as reducing the risk of default. There is therefore an important link between this variable and the credit ratings, as shown in [8]. According to this result, the hypothesis is as follows:

*Hypothesis -3: Board expertise does not affect the credit ratings for the Jordanian firms*.

#### 3.2.4 Board stock

The board compensation plays an important role in attracting and maintaining managers, It permits encouraging managers to perform their duties in the interests of shareholders and ensures a long-term success of the company, [28, 29]. In other words, compensation is an effective incentive for leaders to align their activities with the interests of shareholders. In companies, CEOs and executives prefer less performance-sensitive compensation systems because they run fewer risks of poor performance that would reduce their level of compensation. However, the board of directors prefers to reward managers under a more performance-sensitive compensation contract, such a contract encourages managers to focus on the company’s performance. As a result, a gap of expectation of compensation contract exists between the board and the executives. In order to mitigate the gap of interest, a dialogue between boards and executives should exist to ensure the contract that can be accepted by board and executives. The hypothesis is as follows:

*Hypothesis -4: Board stock does not affect the credit ratings for the Jordanian firms*.

#### 3.2.5 Board size

The board size is an important aspect to be considered because it can influence the performance of the firm. According to management discipline hypothesis, the relationship between shareholders and management can lead to a conflict between them, because of the separation of owners’ equity from the management of the business, which requires better monitoring procedures to regulate this relationship. We note that the size of the board of directors is good for controlling and monitoring the firm’s actions. In our case, the main legislation regulating corporate governance in Jordan is securities law *no. 76/2002*, and firms’ law *no. 22/1997*, for regulating board structure and responsibilities, and the new Listing and Delisting Rules [30] which elaborate the enhancement of board practices within the framework of corporate governance. Public shareholding firms have a single tier board comprising a number of members, with a minimum three and a maximum of thirteen members. The directors must be shareholders. The hypothesis is as follows:

*Hypothesis -5: Board size does not affect the credit ratings for the Jordanian firms*.

## 4 Research design

The aim of this study is to examine the impact of corporate governance variables on the credit ratings for Jordanian listed firms. The dependent variable is credit rating. Credit rate reflects the efficiency of a firm to paying its obligation. As already said, we use eight independent variables consisting of three control variables and five corporate governance variables. In the following we give details about the variables and the methodology used to handle our data.

### 4.1 The dataset description

We work on a dataset from the listed Jordanian firms. All data are available at the following links: Amman Stock Exchange (https://www.ase.com.jo/) and Jordan Securities commission (https://www.jsc.gov.jo/). The direct link is as: https://www.ase.com.jo/ar/history?history_category=64.

We have an annually panel dataset from Jordanian secondary markets. The dataset consists of 576 year-observations of 192 firms recorded in the ASE database for three years from 2015 to 2017 without any missing values. Our data can be divided into two categories: the category 0 that consists of 284 observations having low rates and the category 1 including 292 observations that have high rates. The rating level is issued at the end of each year after the financial report in Jordan.

The annual rating level is computed using an annual financial report. The latter includes financial statements (income statements and balance sheet) issued at the end of each year. This is done only after closing all the financial accounts. These statements summarize the performance of firms during the year. In addition, the financial analysis uses the income statements and balance sheets for firms to assess the firm’s performance.

We note that the assessment of credit rating for a firm is issued by credit rating agencies (CRAs) according to standards given as following:

- The financial solvency of the corporation
- The fulfillment of its previous debts
- The firm or institution’s response to international standards
- The firm’s ability of achieving operational performance that guarantees the rights of creditors and shareholders
- Not achieving a deficit in its balance sheet

Succinctly, all CRAs reflecting their opinions about the firm’s ability to cover service interest and repayment. This is computed by measuring the average of ratings for one year according to financial statements and the above measurement factors. This rating value that represents the likelihood of default risk or late repayments is computed through the examination of the client’s solvency in repaying those loans when they are due. Consequently the aim of credit rating is to determine the client’s merit and its ability to obtain loans and financing according to reporting of CRAs assessments.

In our case, the considered data is rated by World’Vest Base (WVB: http://www.wvb.com/). According to the view of WVB as a rating agency, a firm is given a score value which can be divided into twenty distinct credit rating groups. However, in Jordan companies are classified between *D* to *BB* only. There are about 10 categories. In our case, we divided firms into 4 categories in order to get better results. The classes are represented by letters arrayed downwards from *BB*1 (the best rating) to *D* (payment is in default-bankruptcy). The four categories are given as follows:

*Category 4* which is represented as: (BB: BB1-BB3),
*Category 3* which is represented as: (B: B1-B3),
*Category 2* which is represented as: (C: C1-C3),
*Category 1* which is represented as (D).

Table 1 shows the number of observations for each category when we divided the firms into four categories.

**Table 1:** The dataset description.

WVB Category	Size	WVB Debt Rating	Assigned Rating Score
BB3-BB	176	BB1	4
		BB2	4
		BB3	4
B3-B	219	B1	3
		B2	3
		B3	3
C3-C	55	C1	2
		C2	2
		C3	2
D	126	D	1
**Total**	**576**		

### 4.2 Variables’ measurements

Our model includes eight variables. We have three control variables which are the leverage (Total debt divided by total assets) denoted as LEV, the firm size denoted as SIZE and the Tobin’s q (Growth opportunities) denoted as: TOBQ.

The five corporate governance variables measures are outlined as:

- The board independence (denoted as: BRD_IND) which represents the number of independent directors on the board);
- The role duality (denoted as: R_D) is equals to 1 when the CEO is the chairman at the same time, 0 otherwise;
- The board expertise (denoted as: BRD_EXPERT) which is the number of independent directors that hold seats on other firms’ boards);
- The board stock (denoted as: BRD_STOCK) which is the number of directors that own stock in the firm;
- The board size (denoted as: BRD_SIZE) which is the number of the members on the board.

The dependent variable is the firm’s credit rating (denoted as CR). CR reflects the efficiency of firms.

### 4.3 The used statistical techniques

The statistical techniques can be divided into two main models. The univariate model considers the features as independent Gaussian random variables and the multivariate model that can be used when two or more features are related or correlated.

The ordinal logistic regression (OLR) model is an extension of the binary logistic regression model that has been studied for ordinal multi-class categorization problems [31]. We use OLR to model credit ratings in Jordanian environment. It is an interesting technique for classifying the ordered logit into its constituent components which can add more depth in the data analysis and provide new directions for explanation of the relationships between credit ratings and different group determinants. However, the structure of credit ratings has several econometric issues. The ratings are discrete rather than continuous. Also, there is a natural ordering to the ratings. This means that *AA* is a higher rating than *A*, which is a higher rating than *BBB*. Further, the ratings’ categories are not necessarily evenly spaced. For instance, the *BBB* rating category may traverse a wider range of financial and industry variables than the other categories. More details about this issue can be found in [10].

We use an alternative classification scheme that partitions credit rating (CR) into two categories (*BB* categories), as the indicator of higher credit risk, and lower categories. We compute the marginal changes in the probability of a firm receiving a *BB* category of creditworthiness as a result of a one standardized unit change in each of our governance variables. We use both Logistic Binary Regression (LR) and Ordinal Logit Regression (OLR) models to examine the relation between the credit rating and explanatory variables. The details of our methodology are given in following.

### 4.4 LR and OLR based models

The main objective of this section is to fit ordinal logistic regression (OLR) and binary logistic regression (LR) to our data.

#### 4.4.1 Logistic regression model

We use logistic regression (LR) which is a basic logistic model that estimates the probability that a firm’s credit rating is present or not. LR can be used to predict the behavior of the dependent variable Y using a set of explanatory variables. Binomial or binary logistic regression deals with situations in which the observed outcome for the dependent variable can have only two possible categories: category (1) and non-category (0). For our study, we consider eight explanatory variables (*V*_1_, … *V*_*k*_) where k = 1, …, *p*) and *p* is the number of regression parameters (in our case, *p* = 8).

The explanatory variables are: Leverage, Company size, Tobin’s q, Board independence, Role duality, Board expertise, Board stock and Board size.

Let consider a matrix *X* of *n* rows and *p* columns where *n* is the number of firms and *p* is the number of the explanatory variables.

$$\begin{array}{c} X=[\begin{array}{cccc} X_{11} & X_{12} & \ldots & X_{1p}\\ X_{21} & X_{22} & \ldots & X_{2p}\\ \vdots & \vdots & \ddots & \vdots \\ X_{n1} & X_{n2} & \ldots & X_{np} \end{array}] \end{array}$$

For example, the first row *X*_11_,*X*_12_, …*X*_1*p*_ concerns the specific variables values of the first observation (the firm number 1).

*X*_*ik*_: is the *k*^*th*^ explanatory variable for the observation *i*, where i = 1, .…, *n* and *n* is the sample size. The model can be given as [32]:

$\ln\left[\frac{Pr}{1-Pr}\right]=f(X)+\epsilon =\alpha +>\sum_{k=1}^{p}\left(\beta_{k}X_{ik}\right)+\epsilon ,\text{i}=1,.\ldots ,n$.
Where *n* is the sample size. *β*_*k*_ are a regression coefficient, *ϵ* is the identically and independently distributed error term.
$Pr=\frac{1}{\left(1+\exp\left(-\left[\alpha +\beta_{1}X_{i1}+\ldots \beta_{k}X_{ik}\right]\right)\right)}$
*Pr* represents the probability of being in category 1 and not in category 0 for binary logistic regression.

Since we have two categories of firms, we obtain the *Y*_*i*_ binary response variable where:

*Y*_*i*_ = 1 if the characteristic is present in observation *i*.
*Y*_*i*_ = 0 if the characteristic is not present in observation *i*.

#### 4.4.2 Ordered logit regression model

The Ordered logistic regression (OLR) is a regression model which can be used when we have more than two categories and when the dependent variables are ordered. This model is also known as the proportional odds model (PO). PO model is an ordered logistic model with the assumption that the coefficients for each category are the same. This means that the odds ratio is assumed to be constant for all categories [32]. We apply a similar methodology to the ordinal logistic regression expect that an order ranked list (1, 2, 3, 4) of categories operates and parameter coefficients (*β*) are determined for paired orderings down the risk.

The problem of modelling the credit ratings of firms can be stated as [32]:

Let *Y*_*i*_ be the level of credit rating in each firm *i*,
*X* is the matrix of independent variables,
*J* is the credit rating level (1 = Category 1, 2 = Category 2, 3 = Category 3 and 4 = Category 4).

The firms’ credit rating levels $Y_{ik}^{*}$ can be estimated as follows:

$Y_{ik}^{*}=f(X)+\epsilon =\alpha +\sum_{k=1}^{p}\left(\beta_{k}X_{ik}\right)+\epsilon ,\text{i}=1,\ldots ,n$.
Where *β*_*k*_ are the regression coefficients, *p* = 8, *ϵ* is the identically and independently distributed error term.

Now, let *μ*_*l*_ be the thresholds (cut-offs) for credit rating, *l* = 1, 2, *J* − 1. *J* is the number of categories, Note that the level *l* = 1 represents the minimum threshold, represented as (*D*) in our case.

Since we have four categories of firm, we obtain the following response variables:

Y = 1 represented as (D), if $Y_{i}^{*}\leq \mu_{1}$.
Y = 2 represented as: (C: C1-C3) if *μ*_1_ ≤ $Y_{i}^{*}\leq \mu_{2}$.
Y = 3 represented as: (B: B1-B3) if *μ*_2_ ≤ $Y_{i}^{*}\leq \mu_{3}$.
Y = 4 represented as: (BB: BB1-BB3) if $Y_{i}^{*}>\mu_{3}$.

Since *J* is the number of categories, then the probability of credit rate level (*J*) for a given firm (*i*) can be written as:

$Pr(Y_{i}\leq J)=f(J)=\frac{\exp(\alpha_{J}+\beta_{i}X_{i})}{\left(1+\exp\left(\alpha_{J}+\beta_{i}X_{i}\right)\right)}$.
*Pr*(*Y*_*i*_ = *J*) = *F*(*J*)—*F*(*J* − 1).
Where *β*_*i*_ are the regression coefficients (difference in the log- odds of having credit rate *J* vs. other *J*-1 credit levels), *α*_*J*_ is the intercept for *J*^*th*^ logit.

The current study indicates the effect of corporate governance on credit ratings. Four different categories are used to measure the credit rating. Each category represents successive classes of risk. So the firms are ranked according to their creditworthiness category.

### 4.5 Marginal effects

In the second step of our study, we assess the relative importance of key factors in the above cited models. Their impact can be evaluated by computing the change in probability of receiving a higher rated category arising from corporate governance on firm characteristic variable in turn [9]. These are designated as the marginal effects for this model, which are the direct impacts of a unit change in the respective variable, holding all other variables constant. When we use the logistic regression, the probability of an assessment being in the higher category is as:

$Probability(Y=1)=\frac{oddsratio}{\left(1+oddsratio\right)}$.
Where *oddsratio* = exp(*α* + *β*_1_*X*_*i*1_ + … *β*_*k*_*X*_*ik*_), i = 1, .…, *n*.
*a*, *β*_*k*_ are the respective coefficients.

Therefore, for a unit change in *X*, the partial change in the probability (*Y* = 1) is given by:

$\frac{\hat{\sigma }p}{\hat{\sigma }X}=\frac{\hat{\sigma }}{\hat{\sigma }X}\left[\frac{\exp(a+\beta_{1}X_{i1}+\ldots \beta_{k}X_{ik})}{1+\exp(\alpha +\beta_{1}X_{i1}+\ldots +\beta_{k}Xik)}\right]$
$=\beta *[\frac{\exp(a+\beta_{1}X_{i1}+\beta_{2}X_{i2}+\beta_{3}X_{i3}+\ldots \beta_{k}X_{ik})}{{\left[1+\exp\left(\alpha +\beta_{1}X_{i1}+\ldots +\beta_{k}X_{ik}\right)\right]}^{2}}]=\beta *\text{p}*(1-\text{p}).\text{i}=1,.\ldots ,n$.

The values of *p* and (1-p) are evaluated at their mean values for the independent variables.

The importance of the marginal effects is to assess the sensitivity of the upper credit rating category to a unit change of corporate governance on firms’ characteristic variable. This is important for firm because it shows the key factors and their role to achieve higher grade credit rating.

We should precise that a marginal effect is defined in two ways. First, the marginal effect at the mean (MEM) defined as all variables held at their means. Second, the Average marginal effect (AME) defined as all cases in the sample are then averaging over them.

$\text{MEM}=\frac{\hat{\sigma }p}{\hat{\sigma }X}=g(X\beta )\beta$
$\text{AME}=\frac{\sum_{i=1}^{n}g(X\beta )}{n}*\beta$
Where $g(x\beta )=[\frac{\exp(a+\beta_{1}X_{i1}+\ldots \beta_{k}X_{ik})}{{\left[1+\exp\left(\alpha +\beta_{1}X_{i1}+\ldots +\beta_{k}X_{ik}\right)\right]}^{2}}]$.

The AME is not affected by scale when the variables are normally distributed. Also, the AME works very well in correcting for scaling in Monte Carlo simulations and only a minor bias remains when the variables are strongly skewed. Furthermore, the AME is easy for interpretation. The probability of an average changes by AME points when increases by one unit. Therefore, AME are superior to the popular odds ratios in many respects (robustness, simplicity of interpretation, additivity) (see [33]).

## 5 Empirical results

For both binary logistic regression and ordinal logit regression analysis, two models are tested to examine the impact of both control variables and corporate governance.

### 5.1 A preliminary investigation into the data

The descriptive statistic is presented in this section to describe our data in a simple way. As shown in Table 2, the mean, the standard deviation, the minimum, the maximum, the skewness, and the kurtosis values are given for the four considered categories for different variables.

**Table 2:** Descriptive statistical analysis.

Variables	Categories	Mean	Deviation	Min	Max	Skewness	Kurtosis
BRD_IND	BB3-BB	0.285	0.129	0.000	0.667	0.459	1.332
	B3-B	0.332	0.142	0.000	0.727	0.684	0.997
	C3-C	0.318	0.151	0.000	0.750	0.401	0.258
	D	0.354	0.165	0.000	0.875	0.605	0.799
R_D	BB3-BB	0.230	0.424	0.000	1.000	1.303	-0.310
	B3-B	0.213	0.411	0.000	1.000	1.418	0.011
	C3-C	0.272	0.446	0.000	1.000	1.027	-0.952
	D	0.193	0.395	0.000	1.000	1.567	0.462
BRD_EXPERT	BB3-BB	0.558	0.207	0.077	0.909	0.022	-0.392
	B3-B	0.572	0.200	0.143	1.000	0.370	-0.388
	C3-C	0.524	0.244	0.000	1.429	0.261	-0.048
	D	0.460	0.219	0.000	1.000	0.479	-0.326
BRD_STOCK	BB3-BB	0.751	0.283	0.000	1.000	-1.057	0.001
	B3-B	0.825	0.228	0.099	1.000	-1.537	1.949
	C3-C	0.876	0.215	0.000	1.930	-1.341	6.245
	D	0.873	0.207	0.074	1.930	-0.817	6.005
BRD_SIZE	BB3-BB	9.576	2.457	3.000	13.000	-0.399	-0.213
	B3-B	8.631	2.262	3.000	15.000	0.050	0.165
	C3-C	8.009	2.002	4.000	13.000	0.176	-0.521
	D	7.693	2.177	3.000	13.000	0.319	-0.576

The (BB3-BB) category of board independence has a mean value equals **28.65%** with a standard deviation equals 0.129. The maximum value of this category is 0.667 with a skewness equals 0.684 and a kurtosis equals 1.332. However, the mean value of category (D) for board independence is equal to **35.46%** with a standard deviation equals 0.165. While, the maximum value of category (D) is 0.875 with skewness equals 0.605 and a kurtosis equals 0.799.

Consequently, the (BB3-BB) category of role duality has a mean value equals **23.08%** where a standard deviation is 0.424. The skewness of this category is 1.303 with kurtosis equals -0.310. However, the mean value of category (D) of role duality is equal to **19.32%** with standard deviation equals 39.59, while, the skewness of category (D) is 1.567 with kurtosis of 0.462. Furthermore, the (BB3-BB) category of board expertise has a mean value equals **55.82%** with standard deviation equals 0.207. The skewness of this category is 0.022 with kurtosis equals -0.392. However, the mean value of category (D) of board expertise is equal to **46.04%** with standard deviation equals 0.219, while, the skewness of category (D) is 0.479 with kurtosis equals -0.326.

Moreover, the (BB3-BB) category of board stockholders has a mean value equals **75.12%** with standard deviation equals 0.283. The skewness of this category is -1.057 with kurtosis of 0.001. However, the mean value of category (D) of board stockholders is equal to **87.35%** with standard deviation 0.207, while, the skewness of category (D) is -0.817 with kurtosis of 6.005.

Finally, the (BB3-BB) category of board size has a mean value equals **9.576** with standard deviation equals 2.457. The minimum value of this category is 3 with maximum value of 13, and skewness of this category is -0.399 with kurtosis equals -0.213. However, the mean value of category (D) of board size is equal to **7.693** with standard deviation 2.177, while the while the minimum value of this category is 3 with maximum value equals 13, and the skewness of category (D) is 0.319 with kurtosis equals -0.576.

### 5.2 Endogeneity issues

Endogeneity issues such as omitted variables and reverse causality is an important problem in econometrics and accounting. The problem occurs when we have correlation between the explanatory variables and the error term (epsilon). For instance, this issue comes when the independent variables are measured with error or when the omitted variables confuse the independent and dependent variables. In order to tackle this problem, we conducted several important tests given in the following.

#### 5.2.1 The causality test for all variables

As already said, in our study, we consider a panel dataset. It consists of 192 companies with 3 years for each company. We select control and governance variables carefully from various other variables which are removed based on some tests. First, we remove variables as a result of multicollinearity between independent variables as shown in Table 3. The *“no multicollinearity”* usually refers to the absence of perfect multicollinearity, which is an exact (non-stochastic) linear relation among two or more independent variables [34]. According to the conducted tests, we removed some variables from independent variables which are strong relation with other independent variables. Table 3 gives the correlations between the independent and the dependent variables.

**Table 3:** The correlations between the independent and the dependent variables.

	CR(LR)	CR(OLR)	LEV	SIZE	TOBQ	BRD_IND	R_D	BRD_EXPERT	BRD_STOCK	BRD_SIZE
CR (LR)	1		0.016	0.541	0.159	-0.067	0.065	0.197	0-.159	0.202
CR (OLR)		1	0.034	0.654	0.129	0-.122	0.019	0.167	-0.166	0.266
LEV			1	0.292	-0.073	-0.015	-0.077	0.030	-0.079	0.156
SIZE				1	-0.007	-0.077	0.105	0.180	-0.188	0.377
TOBQ					1	-0.016	-0.020	0.037	-.0056	0.035
BRD_IND						1	0.022	0.087	0.068	0.041
R_D							1	-0.067	0.128	-0.008
BRD_EXPERT								1	-0.008	0.073
BRD_STOCK									1	0.046
BRD_SIZE										1

Note: CR is the dependent variable that represents the credit risk. We run LR and OLR models. In LR model, the dependent variable is binary (0,1) and there are 8 independent variables; control variables and governance variables. On the other hand, the OLR model, the dependent variable is ordinal (1, 2, 3 and 4) and there are also 8 independent variables; control variables and governance variables.

#### 5.2.2 OLS, fixed effects, and random effects models for Endogeneity issues

To illustrate how endogeneity bias may cause incorrect estimates, we examine our proposed model using three different approaches, namely, the ordinary least square (OLS), the fixed effects, and the random effects [35]. The OLS analysis is carried out to examine the direct effect of the independent variables (control variables and governance variables) on the dependent variable (credit rating). The fixed effects is an estimator model that can be used to analyze panel data. It does not allow for the lag of the dependent variables (credit rating) to be included as an explanatory variable in the model [36]. The fixed effects estimation is employed to deal with endogeneity in circumstances where firm-specific characteristics (time invariant) are correlated with the explanatory variable [37]. The random effects model which is called a variance components model can also be used to analyze panel data. Contrary to the fixed effects model, in the random effects model, the individual-specific effect is a random variable that is uncorrelated with the explanatory variables.

To decide between the two models of fixed and random effects, we run a Hausman test where the null hypothesis is that “the preferred model is random effects vs. the alternative is the fixed effects” [38]. This test basically verifies whether the unique errors are correlated with the regressors in the models; the null hypothesis is they are not. If the *p-value* is significant (for example <0.05) then we use the fixed effects model. Otherwise, we use the random effects model. The main result when we run the Hausman test showed that *Chi* − *square* = 9.2043, *df* = 8 and *p* − *value* = 0.3254. Consequently, we don’t reject the null hypothesis *H*_0_: the model is a random effect (because the *p-value* is more than 5%).

However, the Lagrange Multiplier (LM) test helped us to decide between the random effects regression and the simple OLS regression. The null hypothesis in the LM test is that variances across entities is zero. This is no significant difference across units (i.e. no panel effect). The main result of the LM test showed that *Chi* − *square* = 111.92, *df* = 1 and *p* − *value* = 2.2*e*^−16^. Consequently we reject the null hypothesis and conclude that the random effects is the appropriate model for our data. The ordinary least squares regression model fails to control for unobserved heterogeneity and the random-effects model overcomes this problem.

We conclude that within the random effects model, all variables have significant effects on the dependent variables except BRD_STOCK which has a significant effect when we use logistic regression. Table 4 shows the results of the three models: OLS, fixed effects and random effects.

**Table 4:** OLS, fixed effects, and random effect models for Endogeneity issues.

Categories	Variables	OLS		Fixed effects		Random effects	
		Coefficient	Std.Error	Coefficient	Std.Error	Coefficient	Std.Error
Control	Intercept	-7.44946****	0.52907			-6.924858****	0.535826
	LEV	-0.84404****	0.15848	-0.581610***	0.206334	-0.728764****	0.162914
	SIZE	1.33155****	0.07261	1.115114***	0.091317	1.230134****	0.073596
	TOBQ	0.15792****	0.04542	0.142413***	0.051699	0.151548****	0.043734
Governance	BRD_ IND	-0.51850**	0.22398	-0.850242***	0.299070	-0.675708***	0.233408
	BRD_ EXPERT	0.27740*	0.15242	0.338059	0.210215	0.322551**	0.160810
	BRD_ STOCK	-0.11858	0.15386	0.145767	0.180539	0.017807	0.151084
	BRD_ SIZE	0.02420	0.01648	0.050331***	0.018804	0.035698**	0.015904
	R_D	-0.13330	0.08248	-0.127965	0.114944	-0.131280*	0.087423
**Observations**		**576**		**576**		**576**	
**R-square**		**0.4714**		**0.39529**		**0.43161**	
**Adjusted R-square**		**0.4639**		**0.077698**		**0.42359**	
**F-statistic/Chisq**		63.2****		**30.805** ****		432.733****	

Note:

“****”0.001,

“***”0.01,

“**”0.05, and

“*” 0.15

#### 5.2.3 Propensity score matching

In this section, we use the Propensity score matching (PSM) technique to estimate the causal effects of treatments and where the aim is to create a balanced covariate distribution between treated and untreated groups.

The (PSM) technique consists of the folowing main steps [39, 40]: First, we estimate the propensity score using a logistic regression. The logit model generates a binary variable indicating the status of treatments. Table 5 shows the outcome model when applying the logistic regression method. Then we call the matching algorithm and examine the covariate balance in the matched sample. We need a balanced distribution where the covariate distribution is the same within the matched treated and control groups. We use the subclassification matching method with four sub-classes because it is suitable for our larger dataset. As a result, each subclass has approximately the same number of treated units. Table 6 gives a summary of balance for all the data while Table 7 shows the obtained sample sizes by subclasses.

**Table 5:** Outcome model using logistic regression.

**Deviance Residuals:**					**Null deviance**	798.39 on 575 degrees of freedom
**Min**	**1Q**	**Median**	**3Q**	**Max**	**Residual deviance**	538.06 on 567 degrees of freedom
-2.89910	-0.78631	0.06478	0.73974	2.70442	AIC	556.06

	**Coefficients:**			
	**Estimate**	**Std. Error**	** value**	**Pr(> |z|)**
(Intercept)	-25.14591	2.45314	-10.250	< 2e-16 ****
LEV	-2.05894	0.52088	-3.953	7.72e-05 ****
SIZE	3.45925	0.33644	10.282	< 2e-16 ****
TOBQ	0.75071	0.15976	4.699	2.61e-06 ****
BRD_IND	-0.55718	0.69367	-0.803	0.4218
R_D	0.12565	0.25464	0.493	0.6217
BRD_EXPERT	0.90538	0.47639	1.901	0.0574
BRD_STOCK	-0.92877	0.50961	-1.823	0.0684
BRD_SIZE	0.01901	0.05189	0.366	0.7140

Signif. codes: 0 ‘****’ 0.001 ‘***’ 0.01 ‘**’ 0.05 and ‘*’ 0.1 ‘’ 1 (Dispersion parameter for binomial family taken to be 1), Number of Fisher Scoring iterations: 5

**Table 6:** Summary of balance for all data.

	Means Treated	Means Control	Mean Diff	eQQ Med	eQQ Mean	eQQ Max
distance	0.7073	0.3010	0.4063	0.4807	0.4045	0.5268
LEV	0.3209	0.3135	0.0074	0.0140	0.0162	0.0800
SIZE	7.5419	6.9442	0.5977	0.5570	0.5932	1.1100
TOBQ	1.7180	1.4822	0.2358	0.2285	0.2286	0.3930
BRD_IND	0.3168	0.3379	-0.0211	0.0255	0.0359	0.1480
R_D	0.2603	0.2042	0.0560	0.0000	0.0528	1.0000
BRD_EXPERT	0.5626	0.4726	0.0900	0.0960	0.0912	0.4290
BRD_STOCK	0.8129	0.8864	-0.0735	0.0540	0.0765	0.9300
BRD_SIZE	8.6815	7.7746	0.9069	1.0000	0.9085	2.0000

**Table 7:** Sample sizes by subclasses.

	Subclass 1	Subclass 2	Subclass 3	Subclass 4
Treated	73	73	73	73
Control	255	16	8	5
Total	328	89	81	78

The next step is the checking balance procedure where the aim is to measure the balance between the treated and control groups in both the full original dataset, and in the matched dataset. A good matching implies a better balance that should be smaller in the matched dataset compared to the original one. A summary of balance across subclasses is given in Table 8. In Table 9, we give the Percent Balance Improvement denoted four our data.

**Table 8:** Summary of balance across subclasses.

	Means Treated	Means Control	Mean Diff	eQQ Med	eQQ Mean	eQQ Max
distance	0.7073	0.6769	0.0228	0.0415	0.0361	0.0665
LEV	0.3209	0.4226	0.0823	0.0950	0.1356	0.3300
SIZE	7.5419	7.5013	0.0695	0.1591	0.1886	0.4382
TOBQ	1.7180	1.8842	0.2235	0.5286	0.6351	2.0910
BRD_IND	0.3168	0.2906	0.0360	0.0766	0.0936	0.2818
R_D	0.2603	0.1695	0.1043	0.0000	0.1768	0.7500
BRD_EXPERT	0.5626	0.5419	0.0639	0.1133	0.1301	0.3682
BRD_STOCK	0.8129	0.8037	0.0473	0.0685	0.1135	0.5297
BRD_SIZE	8.6815	8.4363	0.3427	0.5000	0.8688	2.5000

**Table 9:** Percent balance improvement.

	Mean Diff.	eQQ Med	eQQ Mean	eQQ Max
distance	92.5279	91.3769	91.0722	87.3765
LEV	-1277.0825	-578.5714	-739.4124	-312.5000
SIZE	93.2111	71.4318	68.2076	60.5180
TOBQ	29.5376	-131.3457	-177.7909	-432.0611
BRD_IND	-24.2147	-200.4902	-160.3462	-90.3716
R_D	-61.8727	0.0000	-234.7374	25.0000
BRD_EXPER	77.0476	-17.9687	-42.5968	14.1608
BRD_STOCK	87.4549	-26.8519	-48.4264	43.0376
BRD_SIZE	72.9581	50.0000	4.3654	-25.0000

**Fig 1:**
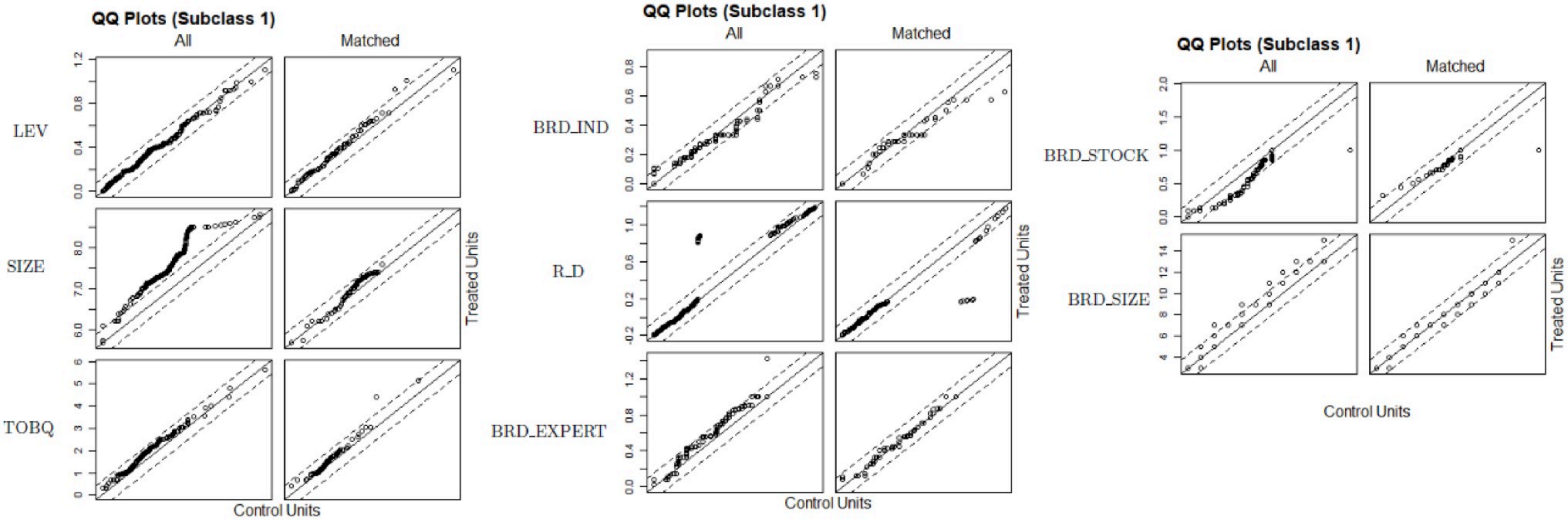
# propensity score locations.

**Fig 2:**
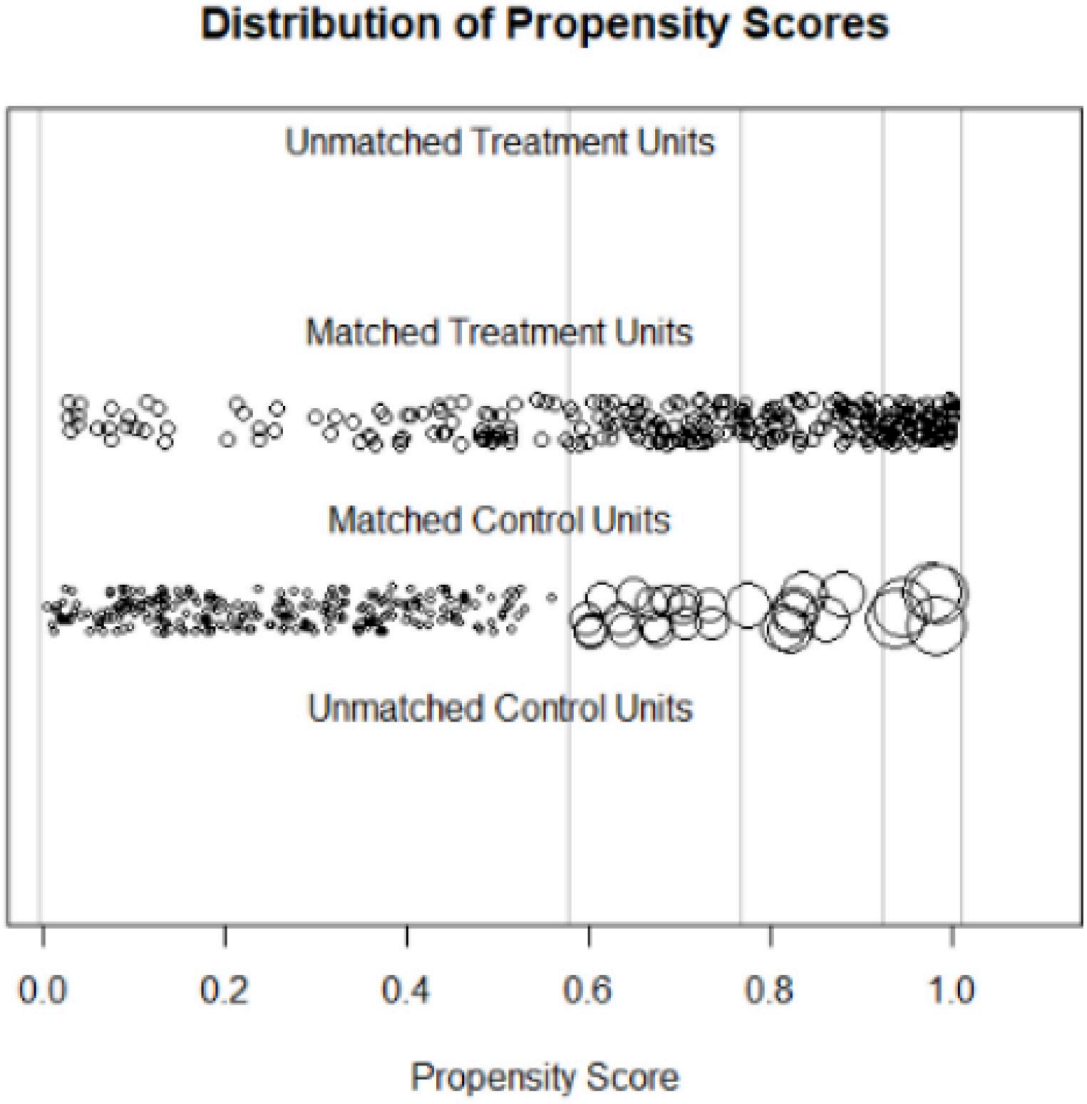
Distribution of propensity scores.

We compute the resulting estimates of the causal treatment effects and draw some descriptive figures. For instance, Fig 1 depicts the propensity score locations for the sub-clas number 1. Fig 2 shows the distribution propensity scores.

**Fig 3:**
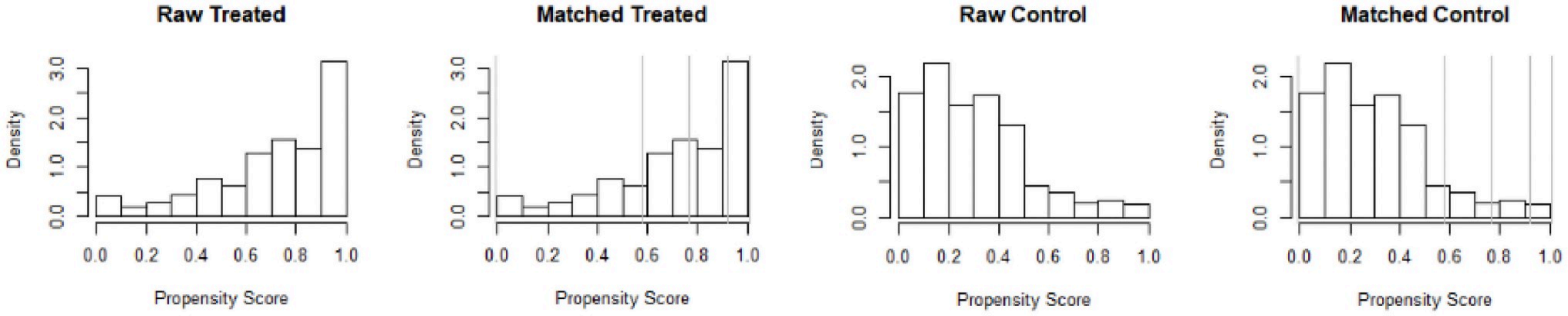
#check matched treated vs matched control.

In Fig 3, we check matched treated vs matched control. From the series of tests, we can conclude that the considered variables are appropriate for our context. This confirms how our findings are robust to the potential endogeneity issue.

### 5.3 The results of LR model

A basic logistic regression uses a binary split, which here reflects a high or a low credit rating. The dependent variable is the probability of a high credit rating as a proportion of the probability of a low credit rating. This is a useful categorization, which will later be extended to incorporate more than two categories. In this basic logistic regression, we investigate the significance of variables in their contribution to the respective credit rating categories. Table 10 gives the results of this model.

**Table 10:** Logistic regression (LR) model.

**Categories**	**Variables**	**Estimated Coefficient**	**S.E**	**t-ratio**	**p-value**
	Constant	-25.212	2.456	10.265	0.000***
Control	LEV	-2.071	0.521	3.975	0.000***
	SIZE	3.465	0.337	10.282	0.000***
	TOBQ	0.759	0.160	4.744	0.000***
Governance	BRD_IND	-0.536	0.693	0.773	0.439
	R_D	0.119	0.255	0.467	0.640
	BRD_EXPERT	0.902	0.476	1.895	0.058**
	BRD_STOCK	-0.917	0.509	1.802	0.072**
	BRD_SIZE	0.020	0.052	0.385	0.707

**Included observations**	**576**
Chi-square (Model)	260.704***
-2Log likelihood	539.047
Cox and Snell R Square	36.4
Nagelkerke R Square	48.5

Note:

*** Significant at the 1%,

** Significant at the 5% and

* Significant at the 11% significance level

The control variables are significant determinants of credit ratings at the **1%** significance level. Regarding to the corporate governance dimension (see Table 10), credit rating is associated positively with board expertise at the **5%** significance level, while board stock is negatively associated with credit rating at the **5%** significance level.

### 5.4 The results of OLR

Table 11 presents the results from an ordered logit regression for the considered variables sample firms. The coefficients obtained from ordered logit regressions are interpreted as follows: a positive (negative) sign means that the dependent variable will move into a higher (lower) category when there is a one unit increase in the independent variable controlling for the other variables in the model. For the second model the adjusted R-square is **51.7%**. This adjusted R-square is reasonably acceptable. As shown in Table 11, the control variables are significantly associated with credit rating at the **1%** significance level. In respect to the OLR model, the corporate governance dimension, credit rating is associated negatively with only Board independent and role duality at the **11%** significance level.

**Table 11:** Ordered logit regression (OLR) model.

**Categories**	**Variables**	**Estimated Coefficient**	**S.E**	**t-ratio**	**p-value**
Control	LEV	-1.755	0.401	4.377	0.000***
	SIZE	4.137	0.268	15.437	0.000***
	TOBQ	0.351	0.124	2.831	0.005***
Governnace	BRD_IND	-0.944	0.563	1.677	0.094*
	R_D	-0.330	0.207	1.594	0.110
	BRD_EXPERT	0.413	0.383	1.078	0.281
	BRD_STOCK	-0.222	0.382	0.581	0.561
	BRD_SIZE	0.030	0.041	0.732	0.467

**Included observations**	**576**
Chi-square (Model)	419.585***
-2Log likelihood	1089.758
Cox and Snell R Square	0.517
Nagelkerke R Square	0.557

Note:

*** Significant at the 1%,

** Significant at the 5% and

* Significant at 11% significance level.

## 6 Conclusion

This paper studied the effects of good corporate governance practices on the firm performance and firm value. The Jordanian regulations and procedures which have recently been introduced to the Jordanian capital market have attracted attention in terms of evaluating the compliance of Jordanian corporations with international corporate governance principles, and application of corporate governance standards on the Jordanian capital market and in the Jordanian economy. In this work, we examined the relationship between corporate governance factors and WVB credit ratings assessment using a sample of Jordanian listed firms. Our models included eight variables. The three control variables were the leverage, the firm size and the Tobin’s *q*. The five corporate governance variables were: the board independent directors, the role duality, the board expertise, the board stock and the board size. We split firms into four categories according to WVB credit rating: from BB1 (the best rating) to D (payment is in default-bankruptcy). Both LR and OLR analysis revealed that the control variables are strong determinants of credit ratings. However, for the governance variables, we found different results. We found that board stockholders and board expertise are moderately significant under LR test, while board independence and role duality are weakly significant under OLR test, board size is insignificant. In general, the empirical analysis showed that the corporate governance variables have a significant impact on credit ratings. Such a finding implies that Jordanian listed firms with a lower proportion of board stock increase the level of their credit ratings. Contrary to agency stewardship theory, management of the Jordanian listed firms with a larger board stock do not necessarily monitor the interests of the shareholders better despite the meeting of interests between them. This leads us to support the role of corporate governance in the determination of credit rating in Jordan. As a future work, we plan to validate our study on other markets and developing countries.
